# PECAM-1 is involved in BCR/ABL signaling and may downregulate imatinib-induced apoptosis of Philadelphia chromosome-positive leukemia cells

**DOI:** 10.3892/ijo.2012.1729

**Published:** 2012-12-06

**Authors:** NAN WU, TETSUYA KUROSU, GAKU OSHIKAWA, TOSHIKAGE NAGAO, OSAMU MIURA

**Affiliations:** Department of Hematology, Graduate School of Medical and Dental Sciences, Tokyo Medical and Dental University, Bunkyoku, Tokyo 113-8519, Japan

**Keywords:** PECAM-1, BCR/ABL, imatinib, apoptosis, leukemia

## Abstract

PECAM-1 (CD31) is an immunoreceptor tyrosine-based inhibitory motif (ITIM)-containing surface glycoprotein expressed on various hematopoietic cells as well as on endothelial cells. PECAM-1 has been shown to play roles in regulation of adhesion, migration and apoptosis. The BCR/ABL fusion tyrosine kinase is expressed in chronic myeloid leukemia and Philadelphia-positive (Ph^+^) acute lymphoblastic leukemia cells, and its inhibition by the clinically used tyrosine kinase inhibitors imatinib or dasatinib induces apoptosis of these cells. In the present study, we demonstrate that PECAM-1 is tyrosine phospho rylated in its ITIM motifs in various BCR/ABL-expressing cells including primary leukemia cells. Studies using imatinib and dasatinib as well as transient expression experiments in 293T cells revealed that PECAM-1 was phosphorylated directly by BCR/ABL, which was enhanced by the imatinib-resistant E255K and T315I mutations, or partly by the Src family tyrosine kinases, including Lyn, which were activated dependently or independently on BCR/ABL. We also demonstrate by using a substrate trapping mutant of SHP2 that tyrosine phosphorylated PECAM-1 binds SHP2 and is a major substrate for this tyrosine phosphatase in BCR/ABL-expressing cells. Overexpression of PECAM-1 in BCR/ABL-expressing cells, including K562 human leukemia cells, enhanced cell adhesion and partially inhibited imatinib-induced apoptosis involving mitochondria depolarization and caspase-3 cleavage, at least partly, in an ITIM-independent manner. These data suggest that PECAM-1 may play a role in regulation of apoptosis as well as adhesion of BCR/ABL-expressing cells to modulate their imatinib sensitivity and would be a possible candidate for therapeutic target in Ph^+^ leukemias.

## Introduction

PECAM-1, also known as CD31, is a 130-kDa glycoprotein member of the immunoglobulin (Ig)-superfamily of type I transmembrane cell adhesion molecules, which is expressed widely on hematopoietic cells as well as on endothelial cells (1,2). PECAM-1 has a 118 amino-acid cytoplasmic tail that contains 2 immunoreceptor tyrosine-based inhibition motifs (ITIMs) that encompass Y663 and Y686 of human PECAM-1. The ITIMs become tyrosine phosphorylated mainly by the Src family tyrosine kinases in response to various stimuli and recruit several SH2-domain containing signaling molecules, including the protein-tyrosine phosphatase SHP2 and the Src family tyrosine kinases. By coupling with these and various other signaling molecules, PECAM-1 is implicated in modulation of intracellular signaling mechanisms regulating a variety of cellular events, including integrin activation, chemotaxis, apoptosis and cell adhesion (1–4). Recent studies on PECAM-1 deficient mice have further revealed that it plays a regulatory role in SDF-1-induced migration of hematopoietic stem cells and megakaryocytes to the bone marrow vascular niche (5,6). The hematopoietic cytokine IL-3 has been shown to induce tyrosine phosphorylation of PECAM-1 in hematopoietic cells (7). However, its significance in the signal transduction mechanisms by which this hematopoietic cytokine regulates proliferation and apoptosis of cells is still unknown. PECAM-1 is also expressed on various types of leukemias, including acute myeloid leukemia (AML) (8), acute lymphoblastic leukemia (ALL) (9), and chronic lymphocytic leukemia (CLL) (10,11), and has been implicated in prognosis of CLL, although it remains controversial. Thus, PECAM-1 is expected to play important roles in regulation of hematopoiesis and in leukemogenesis, possibly through modulation of apoptosis, cell adhesion and migration, although the molecular mechanisms involved have not been explored.

The BCR/ABL fusion protein is encoded by the fusion gene generated by a reciprocal t(9;22) (q34;q11.2) chromosomal translocation causing the Philadelphia chromosome (Ph), which is the molecular signature of chronic myeloid leukemia (CML) and is also observed in 30–40% of ALL (12,13). BCR/ABL is a tyrosine kinase that is constitutively activated and confers survival and proliferation advantages on hematopoietic cells, thus directly contributing to leukemogenesis. CML cells express the p210 form of BCR/ABL, and Ph-positive (Ph^+^) ALL cells mostly express the p190 form, which is generated by the difference in location of gene fusion. Imatinib, a tyrosine kinase inhibitor that blocks the catalytic activity of BCR/ABL, has demonstrated unprecedented efficacy for treatment of CML or Ph^+^ ALL (12–14). However, the resistance to imatinib may develop in significant portions of patients under treatment, especially in those with CML in advanced stages or with Ph^+^ ALL mostly due to the emergence of mutations in the BCR/ABL kinase domain, including the most prevalent E255K and T315I mutations. We previously showed that the E255K or T315I mutant possessed increased *in vitro* kinase activities as well increased ability to induce phosphorylation of itself and several cellular substrates when expressed in COS7 cells or in hematopoietic BaF3 cells as compared with unmutated (native) BCR/ABL (15–17). The increases in transformation abilities for these mutants have also been reported (18,19). The Src family tyrosine kinases are also activated in BCR/ABL-dependent or independent ways and may confer imatinib resistance on these leukemic cells (20–22). To develop more efficient therapeutic strategies against Ph^+^ leukemias, it is very important to gain more insights into the molecular mechanisms involved in imatinib resistance of these leukemias.

In the present study, we show that PECAM-1 is heavily tyrosine phosphorylated on its ITIMs in BCR/ABL-expressing cells, including primary Ph^+^ leukemic cells, at least partly dependent on the BCR/ABL kinase activity. Tyrosine phosphorylated PECAM-1 is physically associated with the SHP2 tyrosine phosphatase and most likely acted as a major substrate for SHP2 in these cells. Intriguingly, tyrosine phosphorylation of PECAM-1 as well as its physical association with SHP2 was enhanced by the imatinib-resistance E255K or T315I mutation. Moreover, overexpression of PECAM-1 enhanced cell adhesion and downregulated imatinib-induced apoptosis on BCR/ABL-expressing hematopoietic cells. These results suggest that PECAM-1 is involved in BCR/ABL-mediated signaling and may enhance the anti-apoptotic effect.

## Materials and methods

### Cells and reagents

A clone of murine IL-3-dependent BaF3 cells transfected with a BCR/ABL cDNA under the control of a tetracycline-inducible promoter, Ton.B210 and the parental control clone, Ton.BaF, were kindly provided by Dr G. Daley (23). Ton.B210 cells were cultured in 10% fetal calf serum (FCS) containing RPMI-1640 medium supplemented either with 10% Wehi3B conditioned medium as the source of IL-3 or with 1 *μ*g/ml doxycycline, which induces the expression of BCR/ABL. Ton.B210/E255K or Ton.B210/T315I cells (16), which inducibly express BCR/ABL with the E255K or T315I mutation, respectively, and 32Dcl3 cells expressing BCR/ABL, Ton.32Dp210 (17), were described previously. The human CML cell line K562 was obtained from the Riken Cell Bank (Ibaraki, Japan). TMD-5 cells, a double Ph^+^ ALL-derived cell line expressing the p190 form of BCR/ABL, were kindly provided by Dr S. Tohda (24). Leukemic blasts were isolated from patients with CML myeloid crisis, Ph^+^ ALL, or Ph^+^ biphenotypic acute leukemia as described previously (16). Written informed consent was provided according to the Declaration of Helsinki, and the study was approved by the ethics committee of Tokyo Medical and Dental University. PLAT-A (25), an amphotropic virus packaging cell line, and 293T (26), a human embryonic kidney cell line, were kindly provided by Dr T. Kitamura and Dr S. Yamaoka, respectively, and maintained in Dulbecco’s modified Eagle’s medium (DMEM) supplemented with 10% FCS.

Imatinib was kindly provided by Novartis (Basel, Switzerland). Dasatinib and sorafenib were purchased from Toronto Research Chemicals Inc. (Toronto, Canada) and LKT Laboratories (St. Paul, MN), respectively. Doxycycline and fibronectin were from Calbiochem (San Diego, CA) and Gibco-BRL (Grand Island, NY), respectively. DiOC_6_ was purchased from Invitrogen (Carlsbad, CA).

Antibodies against PECAM-1 (SC1506), Lyn (SC15), CrkL (SC319), SHP2 (SC280), BCR (SC885), and phospho-Y694-STAT5 (SC9359) were purchased from Santa Cruz Biotechnology (Santa Cruz, CA). An anti-phosphotyrosine monoclonal antibody (4G10, 05-321) as well as antibody against Gab2 (06-967) was purchased from Millipore (Billerica, MA). Antibodies against phospho-Y416-Src (9359) and cleaved caspase-3 (9661) were purchased from Cell Signaling Technology (Beverly, MA). Antibodies against phospho-Y396-Lyn (1645) and β-actin were purchased from Epitomics Inc. (Burlingame, CA) and Sigma, respectively.

### Expression plasmids

Expression plasmids for BCR/ABL, pcDNA3-BCR/ABL, and that for the 56-kDa form of Lyn, pXM-LynA, were described previously (27,28). Retrovirus vectors, pRevTRE and pMIG (Addgene plasmid 9044), were obtained from Clontech (Palo Alto, CA) and Addgene (Cambridge, MA), respectively. pMXs-IG (29) was kindly provided by Dr T. Kitamura. Expression plasmids for wild-type PECAM-1 and its mutant with Y663F and Y686F mutations in the ITIM motives, PECAM-1-ITIM (−), in pcDNA3.0 vector were kindly provided by Dr D. Newman (30,31). The coding regions for wild-type and mutant PECAM-1 were subcloned from these plasmids into retroviral vectors pREV-TRE (*Hin*dIII/*Eco*RV), pMIG (*Eco*RI) and pXMs-IG (*Eco*RI) using the restriction enzymes in parentheses. Expression plasmids for wild-type and substrate-trapping mutant of SHP2, pIRES2-EGF-SHP2-Wt and -DA (Addgene plasmids 12283 and 12286) (32), respectively, were obtained from Addgene. The coding sequences for SHP2-Wt and -DA were excised from these plasmids using *Xho*I and *Sma*I and subcloned into pREV-TRE to give pREV-SHP2-Wt and -DA. An expression plasmid for BCR/ABL, pTetP210, was kindly provided by Dr G. Daley (23). The coding sequence for BCR/ABL was excised from pTetP210 using *Eco*RI and subcloned into pRxZiN obtained from the Riken Gene Bank to give pRxP210.

### Transfection and infection

For transient expression in 293T cells, cells were transfected with indicated plasmids using the Lipofectamine reagent (Invitrogen) according to the manufacturer’s instructions. Cells were harvested 48 h after transfection for immunoprecipitation and immunoblotting.

To obtain BaF3 cells constitutively expressing BCR/ABL, Ton.BaF cells were infected with the recombinant retrovirus obtained from PLAT-A transfected with pRxP210, as described previously (33). Infected cells were cultured in medium lacking IL-3, and a clone expressing BCR/ABL at a high level was selected by limited dilution to give Ton.Bp210-8. This cell line was subsequently transduced again with pRev-PECAM-1, -ITIM (−), or pRevTRE and selected in medium containing hygromycin. Cells were used for subsequent experiments after expression of PECAM-1 or PECAM-1-ITIM (−) was confirmed by immunoblotting. To obtain Ton.B210 cells overexpressing wild-type SHP2 or the D425A mutant, Ton.B210 cells were transduced with pRev-SHP2-Wt or -DA, respectively, and selected in medium containing hygromycin. To obtain Ton.32Dp210 or K562 cells overexpressing PECAM-1 or PECAM-1-ITIM (−), these cells were transduced by the retrovirus vectors in pMXs-IG for pMIG, as described above. GFP-positive cells were sorted by flow cytometry, and expression of PECAM-1 or its mutant as well as BCR/ABL was confirmed by immunoblotting.

### Immunoprecipitation and immunoblotting

Cells were lysed and subjected to immunoprecipitation and immunoblotting as described previously (34). The results shown are representative of experiments repeated at least three times.

### Analyses of cell viability, apoptosis, caspase-3 cleavage, and mitochondrial membrane potential (Δψ_m_)

Cell viability was assessed by counting viable and non-viable cell numbers by the trypan blue dye exclusion method. Flow cytometric analysis of cell cycle and apoptosis was performed as described previously (16). Flow cytometric analysis of caspase-3 cleavage was performed using specific antibodies against cleaved caspase-3 as described previously (17). For analysis of Δψ_m_, cells were stained with DiOC_6_ (Invitrogen) and analyzed by flow cytometry as described previously (16).

### Cell adhesion assay

Adhesion assays were performed essentially as described previously (35,36). In brief, cells were labeled with 5 *μ*M BCECF/AM (Dojindo, Kumamoto, Japan) and plated on wells coated with 5 *μ*g/ml fibronectin for 30 min at 37°C. Adherent cells were measured by Cytofluor II fluorescent plate reader (PerSeptive Biosystems, Foster City, CA). After subtraction of background cell binding to bovine serum albumin-coated wells, the percentage of adherent cells was determined by dividing the fluorescence intensity of the adherent cells by that of the initial cell input.

## Results

### PECAM-1 is tyrosine phosphorylated in primary Ph^+^ leukemic and TMD-5 cells in a manner dependent on the BCR/ABL kinase activity

**Figure 1.**
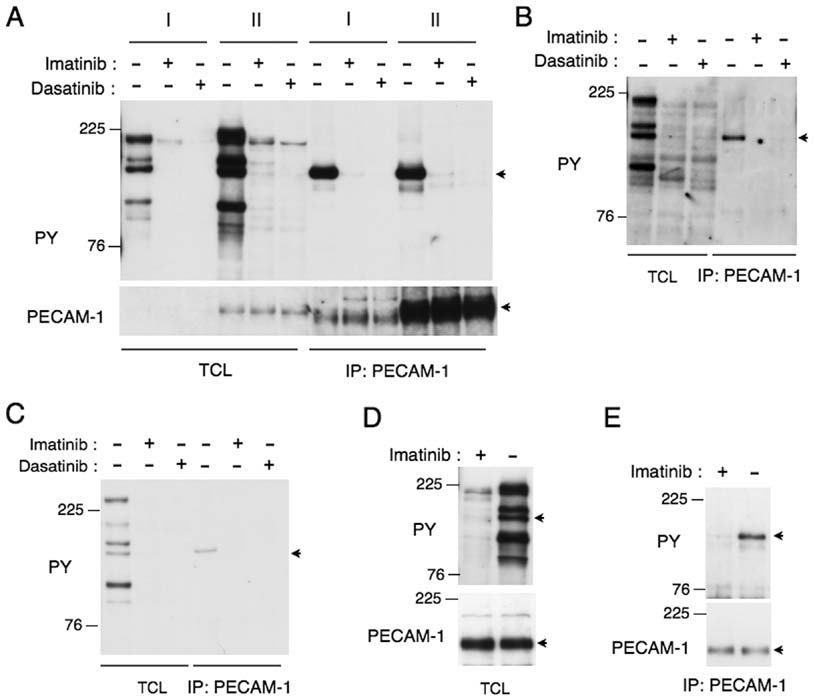
Tyrosine phosphorylation of PECAM-1 in primary Ph^+^ leukemic and TMD-5 cells in a manner dependent on the BCR/ABL kinase activity. (A) Primary leukemic cells from Ph^+^ acute biphenotypic leukemia (I) and ALL (II) patients expressing the p190 form of BCR/ABL were untreated or treated for 2 h with 10 *μ*M imatinib or 0.5 *μ*M dasatinib, as indicated. Cells were lysed and subjected to immunoprecipitation with anti-PECAM-1. Total cell lysates (TCL) and immunoprecipitates were then subjected to western blot analysis using anti-phosphotyrosine (PY) and anti-PECAM-1 antibodies, as indicated. The position of PECAM-1 is indicated by an arrowhead. (B) Primary leukemic cells from a Ph^+^ ALL patient were untreated or treated for 2 h with 5 *μ*M imatinib or 5 nM dasatinib, as indicated and analyzed. (C) Primary leukemic cells from a CML patient in blastic phase were untreated or treated for 2 h with 10 *μ*M imatinib or 500 nM dasatinib, as indicated, and analyzed. (D and E) TMD-5 Ph^+^ ALL cells were treated with 2 *μ*M imatinib for 2 h or left untreated, as indicated. (D) Total cell lysates (TCL) and (E) anti-PECAM-1 immunoprecipitates were analyzed.

To examine possible involvement of PECAM-1 in BCR/ABL-mediated signaling, we first examined whether it is tyrosine phosphorylated in primary Ph^+^ leukemic cells. As shown in Fig. 1A, PECAM-1 was conspicuously phosphorylated on tyrosine in Ph^+^ biphenotypic acute leukemia or ALL cells, which was mostly abolished by treatment with the tyrosine kinase inhibitor imatinib or dasatinib. Essentially the same results were obtained with primary leukemic cells from another patient with Ph^+^ ALL expressing the p190 form of BCR/ABL and a patient with CML in myeloid blastic crisis expressing the p210 form of BCR/ABL (Fig. 1B and C, respectively). We also examined a Ph^+^ ALL cell line expressing the p190 form of BCR/ABL, TMD-5, and found that PECAM-1 was also tyrosine phosphorylated in these cells and was dephosphorylated after treatment with imatinib (Fig. 1D and E). These results suggest that PECAM-1 is a substrate of both p190 and p210 forms of BCR/ABL in these leukemic cells.

### PECAM-1 is tyrosine phosphorylated partly through the Src kinases and is a major substrate of SHP2 in BCR/ABL-expressing cells

**Figure 2.**
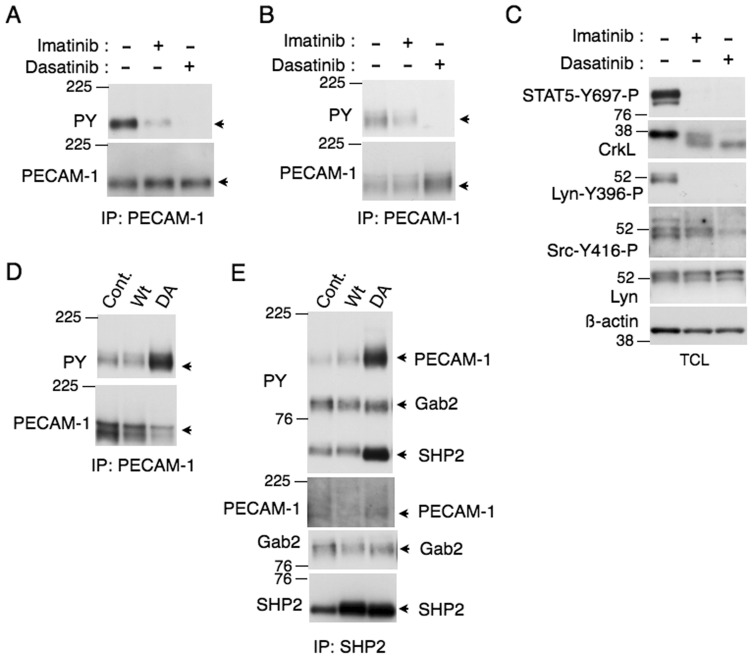
Involvement of BCR/ABL, the Src family kinases and SHP2 in regulation of PECAM-1 tyrosine phosphorylation in various BCR/ABL-expressing cell lines. (A) Ton.32Dp210 cells were treated with 10 *μ*M imatinib or 0.5 *μ*M dasatinib for 6 h or left untreated as control, as indicated, and analyzed. (B and C) Ton. B210 cells, a clone of BaF3 cells inducibly expressing BCR/ABL, were cultured in the presence of doxycycline and treated with 10 *μ*M imatinib or 0.5 *μ*M dasatinib for 2 h or left untreated as control, as indicated. Immunoprecipitates with anti-PECAM-1 or total cell lysates (TCL) were analyzed. (D and E) Ton.B210 cells overexpressing SHP2 (Wt) or its substrate-trapping D425A mutant (DA) and vector-control cells (Cont) were lysed and immunoprecipitated with (D) anti-PECAM-1 or (E) anti-SHP2, as indicated, for analysis.

We next examined murine model hematopoietic cell lines 32Dcl3 and BaF3 engineered to express BCR/ABL. As shown in Fig. 2A and B, PECAM-1 was tyrosine phosphorylated also in these cells, which was completely dephosphorylated by dasatinib. However, imatinib only partially inhibited tyrosine phosphorylation of PECAM-1 in these cells. Because dasatinib, but not imatinib, also inhibits the Src family tyrosine kinases in addition to BCR/ABL, we examined the activation specific tyrosine phosphorylation of these kinases. As shown in Fig. 2C, western blot analysis with an antibody specific for Lyn activation showed that imatinib drastically inhibited activation of Lyn in BCR/ABL-expressing BaF3 cells. However, examination with an antibody that detects activation of the various Src family kinases revealed that some of the activated kinases were resistant to imatinib (Fig. 2C). On the other hand, dasatinib strongly inhibited activation of the Src family kinases including Lyn. These data suggest that Lyn is activated by BCR/ABL in these cells, while some of the other Src family members are constitutively activated independent of BCR/ABL. We also examined the well-established BCR/ABL substrates STAT5 and CrkL in these cells (12). Similar to PECAM-1, tyrosine phosphorylation of CrkL, which was examined by the mobility shift assay, was also abrogated by dasatinib but only partially inhibited by imatinib (Fig. 2C). By contrast, imatinib abrogated tyrosine phosphorylation of STAT5 in BaF3 cells expressing BCR/ABL. These results suggest that the tyrosine phospho rylation of PECAM-1 as well as CrkL, but not that of STAT5, is mediated at least partly by the Src family kinases activated independent of BCR/ABL in these cells.

We next examined the possibility that tyrosine phospho rylated PECAM-1 is a substrate for the SHP2 tyrosine phosphatase in these cells, because SHP2 forms a physical complex with tyrosine phosphorylated PECAM-1 in various types of cells (4,37). For this purpose, we overexpressed wild-type SHP2 or its dominant-negative, substrate-trapping mutant SHP-2-D425A (7,32,38) in Ton.B210 cells. As shown in Fig. 2D and E, overexpression of SHP2-D425A, but not wild-type SHP2, profoundly enhanced tyrosine phosphorylation of PECAM-1 and SHP2. Although SHP2 was found to form a complex with PECAM-1 as well as Gab2 in these cells, tyrosine phosphorylation of PECAM-1, but not that of Gab2, that was associated with SHP2 was drastically enhanced by overexpression of SHP2-D425A (Fig. 2E). Tyrosine phosphorylation of SHP2 was also prominently increased in SHP-2-D425A-expressing Ton.B210 cells. These results suggest that PECAM-1 is a major binding partner and substrate of SHP2 in BCR/ABL-expressing cells.

### PECAM-1 is tyrosine phosphorylated on ITIM by BCR/ABL and Lyn in 293T cells

**Figure 3.**
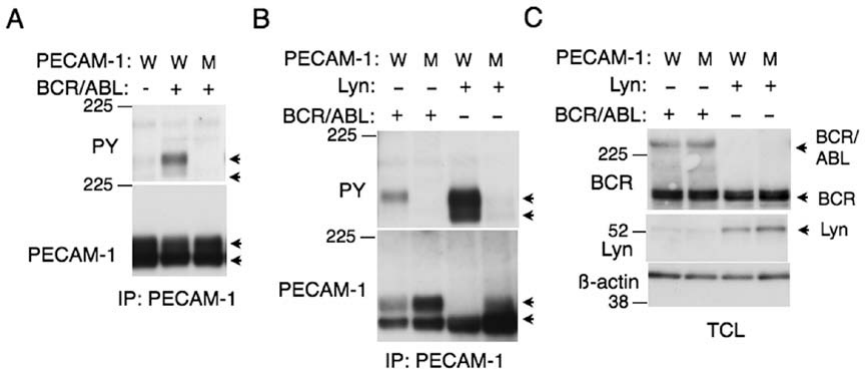
PECAM-1 is tyrosine phosphorylated on ITIMs by BCR/ABL or Lyn in 293T cells. (A) 293T cells were transiently transfected with plasmids coding for BCR/ABL and wild-type PECAM-1 (W) or PECAM-1-ITIM (−) (M), as indicated. Immunoprecipitates with anti-PECAM-1 was analyzed. Positions of PECAM-1 are indicated by arrowheads. (B and C) 293T cells were transfected with plasmids coding for BCR/ABL and Lyn as well as wild-type PECAM-1 (W) or PECAM-1-ITIM (−) (M), as indicated. Immunoprecipitates with anti-PECAM-1 and total cell lysates (TCL) were subjected to western blot analysis using indicated antibodies. Positions of relevant proteins are indicated.

To examine further the mechanisms of tyrosine phosphorylation of PECAM-1 induced by BCR/ABL, we next examined it in transiently transfected 293T cells. As shown in Fig. 3A, co-expression of BCR/ABL induced tyrosine phosphorylation of wild-type PECAM-1 but not that of PECAM-1-ITIM (−) with the mutated ITIM motives (Y663F, Y686F), thus indicating that BCR/ABL induces tyrosine phosphorylation of one or both of these ITIM motives. In accordance with a previous report (39), a smaller 120-kDa form of PECAM-1, which most likely represents a differently glycosylated form, was unambiguously observed in transfected cells.

Because Lyn was activated by BCR/ABL and the Src family kinases were implicated in induction of tyrosine phosphorylation of PECAM-1 in BaF3 cells (Fig. 2B and C), we next examined the ability of Lyn to phosphorylate PECAM-1. When co-expressed in 293T cells, Lyn induced a robust tyrosine phosphorylation of PECAM-1, which was more conspicuously observed than that induced by BCR/ABL (Fig. 3B and C). Lyn also failed to induce phosphorylation of PECAM-1-ITIM (−). These results are consistent with the idea that the Src kinases including Lyn may partly mediate tyrosine phosphorylation of PECAM-1 on the ITIM motives in cells expressing BCR/ABL.

### Tyrosine phosphorylation of PECAM-1 is enhanced by the E255K or T315I imatinib-resistant mutation in BCR/ABL

**Figure 4.**
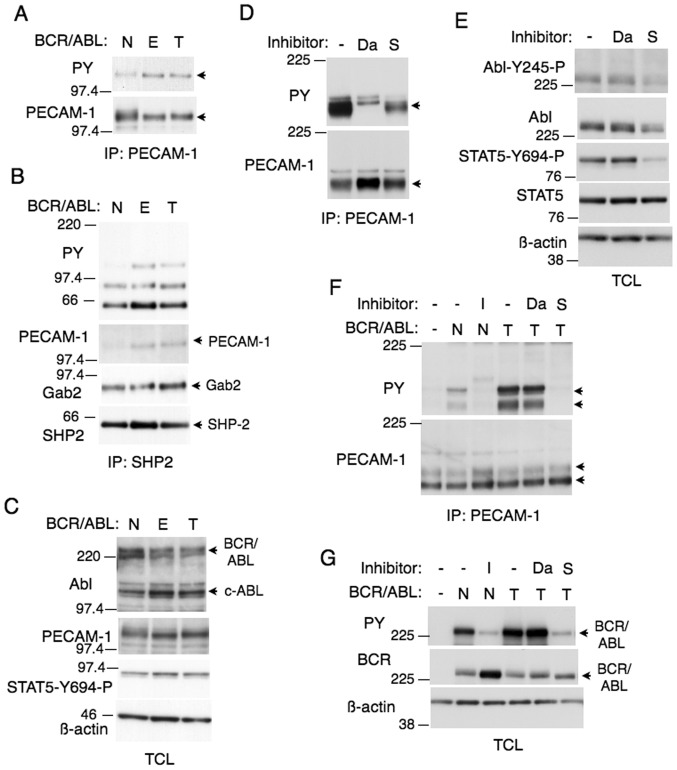
PECAM-1 is more prominently tyrosine phosphorylated in cells expressing the E255K or T315I mutant BCR/ABL. (A, B and C) Ton.B210 (N), Ton.B210/E255K (E), or Ton.B210/T315I (T) cells, cultured in the presence of doxycycline, were lysed. Immunoprecipitates with (A) anti-PECAM-1 or (B) anti-SHP2 as well as (C) total cell lysates (TCL) were subjected to western blot analysis using indicated antibodies. The position of PECAM-1 is indicated by an arrowhead. Positions of other proteins are also indicated. (D and E) Ton.B210/T315I cells were treated with 0.5 *μ*M dasatinib (Da) or 10 *μ*M sorafenib (S) for 2 h or left untreated as control, as indicated, and analyzed. (F and G) 293T cells were transiently transfected with plasmids coding for native BCR/ABL (N) or the T315I mutant (T), as indicated. Cells were treated with 3 *μ*M imatinib (I), 0.5 *μ*M dasatinib (Da), or 10 *μ*M sorafenib (S) or left untreated, as indicated, for 5 h before analysis.

E255K and T315I are the most predominant mutations of BCR/ABL causing imatinib resistance in patients and may increase the kinase activity or change the substrate preferences of BCR/ABL (15,18). Thus, we next examined tyrosine phosphorylation of PECAM-1 in cells expressing BCR/ABL harboring these mutations. As shown in Fig. 4A, PECAM-1 was more prominently tyrosine phosphorylated in BaF3 cells expressing the E255K or T315I mutant as compared with cells expressing native BCR/ABL. Moreover, as shown in Fig. 4B, SHP2 physically associated with PECAM-1 more prominently in cells expressing the E255K or T315I mutant as compared with cells expressing native BCR/ABL, while these mutants had less significant effects on complex formation between SHP2 and Gab2. Tyrosine phosphorylation of STAT5 was also enhanced, though not as significantly as that of PECAM-1, in cells expressing the E255K or T315I mutant (Fig. 4C).

We next examined the effect of dasatinib on tyrosine phosphorylation of PECAM-1 in BaF3 cells expressing the T315I mutant, which is totally resistant to dasatinib as well as imatinib but sensitive to the multi-kinase inhibitor sorafenib (17,22). As shown in Fig. 4D, dasatinib or sorafenib partially inhibited tyrosine phosphorylation of PECAM-1. It was confirmed that sorafenib, in contrast to dasatinib, showed inhibitory effect on autophosphorylation of BCR/ABL or phosphorylation of its substrate STAT5 in these cells (Fig. 4E), thus indicating it partially inhibited the T315I mutant in these cells. In 293T cells, the T315I mutant also induced tyrosine phosphorylation of PECAM-1 much more prominently than native BCR/ABL, which was abolished by sorafenib but not affected by dasatinib and correlated with autophosphorylation of BCR/ABL (Fig. 4F and G). The increase in autophosphorylation of the T315I mutation as compared with native BCR/ABL was not so prominent as compared with that in PECAM-1 phosphorylation. These results suggest that the tyrosine phosphorylation of PECAM-1 is mediated both directly by the T315I BCR/ABL mutant and by the Src family kinases in BaF3 cells and exclusively by BCR/ABL in 293T cells.

### Overexpression of PECAM-1 enhances cell adhesion and downregulates imatinib-induced apoptosis in BCR/ABL-expressing cells in an ITIM-independent manner

**Figure 5.**
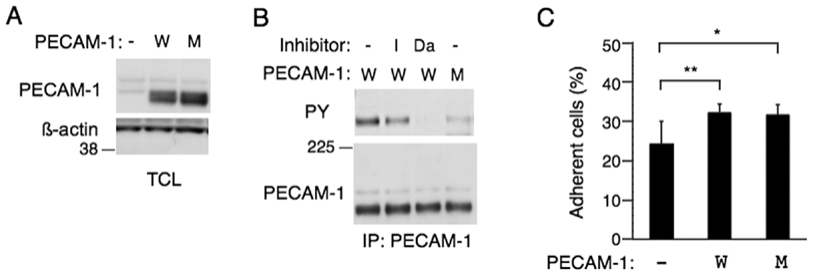
Overexpression of PECAM-1 in human leukemic K562 cells enhances cell adhesion. (A) K562 cells overexpressing wild-type PECAM-1 (W) or PECAM-1-ITIM (−) (M) as well as vector-control cells (−) were lysed. Cellular lysates (TCL) were subjected to western blot analysis with indicated antibodies. (B) K562 cells overexpressing wild-type PECAM-1 (W) or PECAM-1-ITIM (−) (M) were treated for 6 h with 5 *μ*M imatinib (I) or 10 nM dasatinib (Da) or left untreated (−), as indicated, and immunoprecipitates with anti-PECAM-1 were analyzed. The position of PECAM-1 is indicated by an arrowhead. (C) K562 cells overexpressing wild-type PECAM-1 (W) or PECAM-1-ITIM (−) (M) as well as vector-control cells (−) were fluorescently labeled and allowed to adhere to wells coated with 5 *μ*g/ml fibronectin for 30 min. The extent of cell adhesion was quantitated as described in Materials and methods. The data represent the mean ± SD and representative of experiments repeated three times. The asterisks indicate statistically significant differences determined by Student’s t-test [^*^p<0.05, ^**^p<0.01 (n=6)].

To examine the cellular effects of PECAM-1 in Ph^+^ leukemic cells, we next examined the human CML K562 cell line, which expresses endogenous PECAM-1 at a barely detectable level (Fig. 5A). As in other BCR/ABL-expressing cells, PECAM-1 overexpressed in K562 cells was tyrosine phosphorylated, which was moderately inhibited or abolished by imatinib or dasatinib, respectively (Fig. 5B). On the other hand, K562 cells overexpressing PECAM-1-ITIM (−) showed a very low level of PECAM-1 phosphorylation, thus suggesting that the ITIM motives are mainly tyrosine phosphorylated also in these Ph^+^ leukemic cells.

We first examined the possible effect of PECAM-1 on cell adhesion. As shown in Fig. 5C, adhesion of K562 to fibronectin-coated plate was enhanced by overexpression of PECAM-1 or PECAM-1-ITIM (−). These data are in agreement with the previous reports that PECAM-1 may play a role in regulation of integrin activation and cell adhesion in various cell types (3,37).

**Figure 6.**
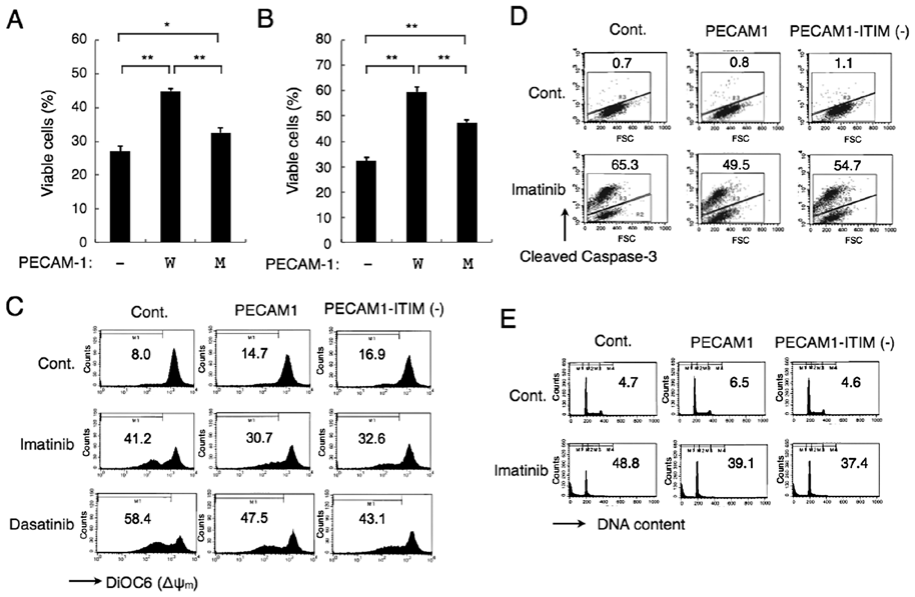
Overexpression of PECAM-1 partially inhibits imatinib-induced apoptosis in BCR/ABL-expressing cells in an ITIM-independent manner. (A and B) BCR/ABL-expressing 32D (A) or K562 (B) cells overexpressing wild-type PECAM-1 (W) or PECAM-1-ITIM (−) (M) as well as vector-control cells at 1×10^5^/ml were cultured with 3 *μ*M imatinib for (A) 36 or (B) 48 h, and viability was determined after trypan blue staining. The data represent the mean ± SD of triplicate samples and representative of experiments repeated three times. ^*^p<0.05, ^**^p<0.005 (n=3). (C) K562 cells overexpressing PECAM-1 or PECAM-1-ITIM (−) and vector-control cells were treated for 36 h with 0.6 *μ*M imatinib or 2 nM dasatinib or left untreated, as indicated, and analyzed for Δψ_m_ by flow cytometry using DiOC_6_. Percentages of cells showing loss of Δψ_m_ are indicated. (D) K562 cells indicated were treated for 36 h with 0.6 *μ*M imatinib or left untreated, as indicated, and analyzed for cleavage of caspase-3 by flow cytometry. Percentages of cells showing cleavage of caspase-3 are indicated. (E) K562 cells indicated were treated for 36 h with 3 *μ*M imatinib or left untreated, as indicated, and analyzed for cellular DNA content by flow cytometry. Percentages of apoptotic cells sub-G1 DNA content are indicated.

We next examined the possibility that PECAM-1 may affect the sensitivity of Ph^+^ leukemic cells to the tyrosine kinase inhibitors, because PECAM-1 has been implicated in prevention of apoptosis in various types of cells (40–44). As shown in Fig. 6A and B, the decline in viability induced by imatinib was downregulated by overexpression of PECAM-1 or, to a lesser degree, by that of PECAM-1-ITIM (−) in BCR/ABL-expressing 32D cells or in K562 cells, respectively. Moreover, PECAM-1 or its mutant was found to partially prevent depolarization of Δψ_m_ in K562 cells treated with imatinib or dasatinib (Fig. 6C). In accordance with this, PECAM-1 also partially protected K562 cells from imatinib-induced cleavage of caspase-3 (Fig. 6D). It was finally confirmed that PECAM-1 or its mutant decreased the number of K562 cells with sub-G1 DNA content, a hallmark of cells undergoing apoptosis, after treatment with imatinib (Fig. 6E). Similarly, overexpression of PECAM-1 or its mutant in 32D cells partially downregulated depolarization of Δψ_m_, cleavage of caspase-3, and appearance of cells with sub-G1 DNA content induced by imatinib (data not shown). Overexpression of PECAM-1 and its mutant showed comparable anti-apoptotic effects in these repeated experiments in K562 and 32D cells. These data suggest that PECAM-1 may partially protect BCR/ABL-expressing leukemic cells treated with the tyrosine kinase inhibitors from activation of mitochondria-mediated apoptotic pathway leading to caspase activation and DNA fragmentation, at least partly, in an ITIM-independent manner.

## Discussion

We have found that PECAM-1 was heavily tyrosine phosphorylated in all the four Ph^+^ leukemic blast samples we examined, including those from biphenotypic AL, ALL and CML-BC patients, the effect was drastically inhibited by imatinib, thus indicating its phosphorylation was mediated by BCR/ABL (Fig. 1). Further studies indicated that tyrosine phosphorylation of PECAM-1 was exclusively mediated by BCR/ABL in 293T cells but was additionally mediated through the Src family kinases, including Lyn, that are activated constitutively or by BCR/ABL in various hematopoietic cell lines, such as BaF3, 32D and K562 cells (Figs. 2–5). In this regard, it is noteworthy that BCR/ABL-independent activation of Lyn has been implicated in development of imatinib-resistance in patients with mutation-negative BCR/ABL (21). Although tyrosine phosphorylation of PECAM-1 ITIMs *per se* may not be required for the anti-apoptotic effect of PECAM-1 in imatinib-treated leukemic cells as discussed below, it is tempting to speculate that interaction of Lyn with PECAM-1 might be involved in acquisition of resistance in these cases, which needs to be addressed in future studies. Previous studies have shown that PECAM-1 is expressed in various types of leukemic cells and implicated its expression in development of the central nervous system involvement of ALL (9), emigration of AML cells from the bone marrow by transendothelial migration (8), and in determination of prognosis of CLL (10). However, the tyrosine phosphorylation status of PECAM-1 has not been examined in these leukemic cells. Thus, its examination in various leukemic cells may shed more light on the significance of PECAM-1 in pathogenesis and prognosis of leukemias.

It is well established that PECAM-1 recruits SHP2 through interaction between its tyrosine phosphorylated ITIMs and the SH2 domains of the tyrosine phosphatase and activates its phosphatase activity (3,4,37). In agreement with this, we observed that tyrosine phosphorylated PECAM-1 formed a complex with SHP2 in BCR/ABL-expressing hematopoietic cells (Fig. 4B). Furthermore, by using the substrate-trapping, loss-of-function mutant of SHP2, we revealed that PECAM-1 is a major substrate of SHP2 in these cells, because PECAM-1 represented an SHP2-associated tyrosine phosphorylated protein that was most significantly enhanced by introduction of SHP2-D425A in BaF3 cells expressing BCR/ABL (Fig. 2E). Previously, Wheadon *et al*(7) showed that tyrosine phosphorylation of PECAM-1 and Gab2 that bound SHP2 was significantly increased by overexpression of a substrate-trapping C459S mutant of SHP2 in BaF3 cells stimulated with IL-3, thus indicating these proteins are substrates of SHP2 in these cells. Somewhat different from their results, we found that tyrosine phosphorylation of Gab2 that bound SHP2 was not significantly enhanced by overexpression of SHP2-D425A as compared with that of PECAM-1. Therefore, although BCR/ABL and the IL-3 receptor activate similar signaling events involving SHP2 and Gab2, PECAM-1 may play a relatively more significant role as an SHP2-binding substrate as compared with Gab2 in signaling events downstream of BCR/ABL. Previously, SHP2 was postulated to be activated by binding with Gab2 and to play a crucial role in leukemogenesis by BCR/ABL (45–47). The present study raises a possibility that PECAM-1 may also play an important role in activation of the SHP2 signaling events downstream of BCR/ABL.

Intriguingly, the imatinib-resistant BCR/ABL mutants E255K and T315I showed increased ability as compared with native BCR/ABL to induce tyrosine phosphorylation of PECAM-1 when expressed in the murine model hematopoietic cell line BaF3 cells or in 293T cells (Fig. 4). Furthermore, the E255K and T315I mutations enhanced the complex formation between PECAM-1 and SHP2 (Fig. 4B). The enhanced tyrosine phosphorylation of PECAM-1 by the E255K and T315I mutants may be at least partly due to their enhanced activities (15–17). However, as compared with tyrosine phosphorylation of BCR/ABL and STAT5, that of PECAM-1 was more significantly enhanced by these mutations (Fig. 4A, C, F and G). In this regard, it was previously reported that the imatinib-resistant mutants, including E255K and T315I, exhibited different patterns of substrate phosphorylation as compared with native BCR/ABL, thus suggesting altered substrate specificity and pathway activation (18). Therefore, it is possible that the E255K and T315I mutants may more efficiently interact with and phosphorylate PECAM-1 as compared with native BCR/ABL. Because these mutants are endowed with not only imatinib resistance but also enhanced transforming activities (18,19), a possible significance of PECAM-1 in transforming mechanisms for BCR/ABL including these mutants needs to be addressed in future studies.

The present study revealed that PECAM-1 may enhance the anti-apoptotic effect of BCR/ABL and partially confer imatinib resistance on BCR/ABL-expressing cells by inhibiting the mitochondria-mediated apoptotic mechanisms in a manner at least partly independent of phosphorylation of ITIMs (Fig. 6). Anti-apoptotic effects of PECAM-1 have been previously reported for several types of cells under various conditions, including endothelial cells withdrawn from serum (41,44), hematopoietic cells withdrawn from GM-CSF (42) and an ALL cell line treated with UV irradiation or DNA-damaging chemotherapeutic agents VP16 and AraC (40,43). It was also found that PECAM-1 prevented mitochondria-dependent apoptosis of HEK293T cells induced by overexpression of Bax (43). In these studies, involvement of its tyrosine phosphorylation and binding with SHP2 in anti-apoptotic effects has been controversial (40,43,44). Moreover, activation of the PI3K/Akt signaling pathway with upregulation of anti-apoptotic Bcl-2 and Bcl-Xl expression has been implicated in some of these reports (41,42,44), but not in others (40,43). Thus, it is speculated that anti-apoptotic effects of PECAM-1 are mediated through several different mechanisms in different types of cells under various apoptotic stimuli. Intriguingly, PECAM-1 was reported to bind tyrosine-phosphorylated β-catenin, which was independent of tyrosine phosphorylation of PECAM-1, and to affect its degradation through GSK3β-mediated degradation (48). It was also reported that β-catenin is stabilized through tyrosine phosphorylation by BCR/ABL and may play an essential role in survival of leukemic stem cells expressing BCR/ABL (49–51). Further studies are in progress in our laboratory to address the possible involvement of β-catenin in PECAM-1-mediated anti-apoptotic mechanisms independent of ITIM phospho rylation. It is also notable that overexpression of PECAM-1 or PECAM-1-ITIM (−) in K562 cells enhanced adhesion of these cells to fibronectin (Fig. 5C), because cell adhesion has been strongly implicated in survival and drug resistance of leukemic cells (52). Taken together with the previous report implicating PECAM-1 in migration of hematopoietic cells to the bone marrow niche (6), where adhesion as well as soluble factors mediate prosurvival effects (52), it is possible that PECAM-1 may play a more prominent role in protection of Ph^+^ leukemic cells from apoptosis in patients treated with imatinib than that expected from the present study. Further studies are warranted to address these possibilities and to elucidate the mechanisms underlying the anti-apoptotic effect of PECAM-1 in Ph^+^ leukemic cells.
